# Gains v. losses, or context dependence generated by confusion?

**DOI:** 10.1007/s10071-019-01339-1

**Published:** 2020-01-21

**Authors:** Alasdair I. Houston, Karoline Wiesner

**Affiliations:** 1 School of Biological Sciences, Bristol Life Sciences Building, University of Bristol, Tyndall Avenue, Bristol, BS8 1TQ UK; 2 School of Mathematics, University of Bristol, University Walk, Bristol, BS8 1TW UK

**Keywords:** Framing, Scalar utility theory

## Abstract

Tversky and Kahneman introduced the term framing for the finding that people give different answers to the same question depending on the way it is posed. One form of framing involves presenting the same outcome as either a gain or a loss. An experiment on starlings by Marsh and Kacelnik suggests that this form of framing occurs in non-humans. We argue that the experimental result demonstrates framing in the general sense of context dependence but does not provide compelling evidence of framing in terms of gains and losses. A version of scalar utility theory which is extended to include the possibility of memory errors accounts for the data and suggests future lines of research.

## Introduction

It is well established that the way in which a problem is worded influences the answer that people give, see Kahneman and Tversky (1984), Tversky and Kahneman (1981, 1986), Levin et al. (1998). Tversky and Kahneman introduce the term “framing” as a name for this effect (Tversky and Kahneman 1981). A defining feature of a framing experiment is that the choices available to an animal, and their consequences, do not change but behaviour does (Tversky and Kahneman 1981; Kühberger 1998). Although framing is often discussed in relation to prospect theory (Tversky and Kahneman 1981; Kühberger et al. 1999; Schneider 1992; Mishra et al. 2012; Ganegoda and Folger 2015), it is defined in terms of behaviour and could be brought about by various mechanisms (e.g. Tombu and Mandel 2015; Marsh and Kacelnik 2002; Schneider 1992; Kühberger 1997).

Given the widespread interest in evaluating whether the cognitive abilities of humans and non-humans are similar, see Ludvig et al. (2014), Penn et al. (2008), Premack (2007), Shettleworth (2012); Santos and Rosati (2015), the existence of framing in non-humans has been investigated. Some framing experiments on humans involve describing the same outcome as either a gain or a loss (e.g. Kahneman and Tversky 1984; Tversky and Kahneman 1981, 1986; Levin et al. 1998; Kühberger 1998; Kühberger et al. 1999; Schneider 1992; Mishra et al. 2012). Marsh and Kacelnik describe an experiment on starlings (*Sturnus vulgaris*) that they consider to provide an example of this form of framing (Marsh and Kacelnik 2002). Each of 14 starlings was presented with a panel with 3 circular keys. After ten pecks on the central key, both of the side keys were illuminated. In choice trials, the left and right keys were illuminated with different symbols. Each symbol was associated with a particular outcome. If the starling pecked the key with one symbol, it always obtained four pellets of food. If it pecked the key with the other symbol, it obtained either two pellets or six pellets of food with equal probability. Thus, each option gave the same mean amount of food, but one was constant (fixed) and the other was variable. In standard trials, the left and right keys were illuminated with the same symbol. There were two treatments. In one (the “gains treatment”), both options gave one pellet, whereas in the other (the “losses treatment”), both options gave seven pellets. During tests, 25 % of trials were choice trials. The argument of Marsh and Kacelnik is that the standard trials frame the outcomes of choice trials. In the “gains treatment”, the mean amount on choice trials is greater than the amount obtained on standard trials and in the “losses treatment”, the mean amount on choice trials is less than the amount obtained on standard trials. From this, Marsh and Kacelnik argue that the outcomes are framed as gains in the former treatment and losses in the latter. Such a claim goes beyond the effect of treatment to argue for a particular way in which the effect occurs. Our view is that although the effect of treatment demonstrates framing, the results can be explained without assuming that the outcomes are framed as gains or losses.

Marsh and Kacelnik discuss three approaches to explaining which option is chosen. Risk-sensitive foraging theory (e.g. McNamara and Houston 1992) is based on linking food to evolutionary success. If the value of food shows diminishing returns, then the constant option should be preferred. Prospect theory (e.g. Kahneman and Tversky 1979) also involves a function that scales outcomes, but the function is different for gains and losses and results in preferring constant gains and variable losses. Although both prospect theory and risk-sensitive foraging theory involve rescaling outcomes, they are fundamentally different in that unlike risk-sensitive foraging theory, prospect theory produces violations of rationality (Houston et al. 2014). The approach that Marsh and Kacelnik concentrate on is scalar utility theory (SUT) (see e.g. Kacelnik and Brito e Abreu 1998; Kacelnik and El Mouden 2013; Rosenström et al. 2016). Instead of being based on assigning value to outcomes, SUT is based on how they are perceived. We now summarise the way in which Marsh and Kacelnik use SUT to predict the results of their experiment.

At the heart of SUT is the idea that an animal does not perceive its environment accurately. Instead it makes systematic errors, in that the standard deviation of the error of each perceived variable is proportional to its mean (this is the scalar property), so that the coefficient of variation is constant. This scalar property of the theory is based on Ernst Heinrich Weber’s concept of a “just noticeable difference”. Approaches based on the scalar property have been used in the study of spatial and temporal behaviour (e.g. Gibbon 1977; Cheng 1992; Cheng et al. 1999; Kacelnik and Brunner 2002; Lejeune and Wearden 2006). For a general review, see Akre and Johnsen (2014). In the model of Marsh and Kacelnik, the coefficient of variation is the only parameter. The animal builds up an internal representation of each option based on its perception of the consequences of choosing the option (e.g. the amount of food obtained). When it is faced with a choice between options, the animal draws a sample from memory for each option. If the animal is trying to select the option that provides the largest amount of food, it will choose the option with the biggest sample. Reboreda and Kacelnik presented starlings with a choice between a fixed option and a variable option with two equiprobable outcomes having the same mean as the fixed option (Reboreda and Kacelnik 1991). Under these conditions, SUT predicts a preference for the fixed option, i.e. risk aversion. This effect does not depend on drawing a single sample (Kacelnik and Brito e Abreu 1998). Marsh and Kacelnik apply SUT to their experiment by claiming that the above argument applies to the “gains treatment”, whereas in the “losses treatment”, a starling will want to minimise its loss and hence select the option with the smaller sample. In other words, the starlings are predicted to be risk-averse in the gains treatment and risk-prone in the losses treatment. Marsh and Kacelnik found that in the gains treatment, 8 out of 14 starlings preferred the fixed option [not significant], whereas in the losses treatment 12 of 14 preferred the variable option [significant], i.e. the SUT prediction was supported in the “losses treatment”.

We are not convinced by the attempt of Marsh and Kacelnik to explain their data in terms of a version of SUT. As they point out [p 3354], in both treatments, the choice outcomes yield a gain in absolute terms. Starlings should choose the option associated with the most food in each treatment, and hence risk-prone behaviour should not be found. We argue that the effect of treatment can be predicted from a version of SUT that includes the possibility of errors in memory.

In the following, we introduce the new mathematical model based on SUT, extended by a parameter quantifying the errors in memory. We present simulated data for various values of this memory parameter and compare our results to the experimental findings by Marsh and Kacelnik. The new model predicts the behaviour qualitatively, and even quantitatively to a small degree.

## Model

According to SUT, an alternative with a fixed outcome forms an internal representation of a normal distribution with a mean $\mu$ equal to the actual outcome. If an alternative has a set of different outcomes, the internal representation takes the form of a mixture of normal distributions with means equal to the respective outcomes and weighted by their respective probabilities. Furthermore, the standard deviation of these distributions increases linearly with the mean, $\sigma = \gamma \mu$. The proportionality constant $\gamma$ is the coefficient of variation. Thus, memories of larger outcomes are subject to a larger error than memories of smaller outcomes.

We represent an option with outcome *a* by a normal distribution $\mathcal {N}_a$ with mean $\mu = a$ and standard deviation $\sigma = \gamma a$. The probability of an option with outcome *a* being judged preferable to an option with outcome *b* is then equal to the probability $\Pr (m_a > m_b)$ of a sample $m_a$ from distribution $\mathcal {N}_a$ being bigger than a sample $m_b$ from distribution $\mathcal {N}_b$. This probability is equal to the cumulative of the normal distribution $\mathcal {N}_{b{-}a}$ with mean $\mu = b-a$ and standard deviation $\sigma = \sqrt{(\gamma a)^2 + (\gamma b)^2}$,

1 $$\begin{aligned} \Pr (m_a > m_b) = \int _{-\infty }^0 \mathcal {N}_{b-a}(t) \mathrm{d}t =: \varPhi _{b-a} . \end{aligned}$$

Before we introduce a version of SUT extended by an error in memory, let us consider a trial with one variable option $$S_V$$ with outcomes *a* or *b*, and a fixed option $$S_F$$ with outcome *f*. The outcomes *a* and *b* of the variable option have probability $$p_a$$ and $$p_b = 1-p_a $$, respectively. According to SUT, the animal, faced with the choice between a fixed and a variable option, retrieves a sample $$m_f$$ from its cognitive representation of $$S_\mathrm{F}$$—the normal distribution $$\mathcal {N}_f$$—and compares it to a memory sample $$m_v$$ of the cognitive representation of the variable alternative $$S_\mathrm{V}$$. With probability $$p_a$$, the memory sample $$m_v$$ of $$S_\mathrm{V}$$ will be taken from distribution $$\mathcal {N}_a$$ and with probability $$p_b$$ it will be taken from $$\mathcal {N}_b$$. Hence, the probability that the fixed outcome is judged preferable to the variable outcome depends on which memory is recalled. This probability is now a weighted sum of the probability of $$m_f$$ being judged preferable to $$m_a$$ (which is the cumulative probability $$\phi _{a-f}$$) and the probability of $$m_f$$ being judged preferable to $$m_b$$ (which is the cumulative probability $$\phi _{b-f}$$). We can write the probability of the fixed alternative (with outcome *f*) being judged preferable to the variable alternative using the cumulative distribution, as a weighted sum:

2 $$\begin{aligned} \Pr (m_f > m_{v}) = p_a \varPhi _{a-f} + p_b \varPhi _{b-f}. \end{aligned}$$

We now turn to the experimental setup of choice and standard trials with starlings used by Marsh and Kacelnik (2002). The settings of the choice and the standard trials are identical and only differ in the symbols on the choice keys. This leads us to propose that the animals do not perfectly distinguish the “framing” standard trial from the choice trial. Rather, we suggest that in their confusion the starlings make decisions based on a mixed memory of both.

To reflect such a confusion mathematically, we extend SUT by a memory parameter, $\theta$. This parameter weighs the influence of the memory of the standard trial on the decision in the choice trial. In other words, $\theta$ accounts for the extent of the confusion. An animal, faced with a choice between a fixed and a variable option, draws a sample from memory as described above. With probability $\theta$, however, it retrieves a sample from the wrong memory, that is in this case the memory of the standard trial. We now modify Eqs. 1 and 2 to account for this confusion. Beginning with Eq. 1, we obtain the following generalised expression for the probability of some fixed option with outcome *a* being judged preferable to some other fixed option with outcome *b*:

3 $$\begin{aligned} \Pr (m_a > m_b | \theta ) = \theta ^2 \varPhi _{{s} - {s}} + \theta (1-\theta ) \left[ \varPhi _{{s} - a} + \varPhi _{b - {s}} \right] + (1-\theta )^2 \varPhi _{b - a} \, , \end{aligned}$$

where we denote the outcome of the standard option with ‘s’. Here, $\phi _{s-s}$ is the (trivial) case of Eq. 1 for two equal options $a=b$, which yields, by definition, $\phi _{s-s} = 1/2$. With probability $\theta$ either *a* or *b* is confused with the standard option and with probability $\theta ^2$ both are confused with the standard option. Thus, for $\theta = 0$, the memory of the standard trial is never sampled and we recover Eq. 1. For $\theta = 1$, the memory of the standard trial is exclusively sampled and none of the options of the current trial are actually considered. As a result, the probability that outcome *a* is preferred over outcome *b* is 1/2, independent of the values of *a* and *b*.

Next, we generalise the expression for the probability of preference with one option being variable (Eq. 2). For a choice trial with one fixed option (outcome *f*) and one variable option (outcomes *a* or *b*) and a standard trial (outcome *s*), Eq. 2 becomes:

4 $$\begin{aligned} \begin{aligned}&\Pr (m_f > m_{v} | \theta ) = \theta ^2 \varPhi _{{s} - {s}} \\&\quad + \theta (1-\theta ) \left[ \varPhi _{{s}-f} + p_a \varPhi _{a - {s} } + p_b \varPhi _{b - {s}} \right] \\&\quad + (1-\theta )^2 \left[ p_a \varPhi _{a-f} + p_b \varPhi _{b-f} \right] \,. \end{aligned} \end{aligned}$$

Note that the model described here has two parameters only, the memory parameter $\theta$ and the coefficient of variation $\gamma = \sigma / \mu$. In the next section, we present predictions from Eq. 4 for various values of $\theta$ and $\gamma$ and compare the results to the experimental findings on starlings by Marsh and Kacelnik.

## Results

**Fig. 1:**
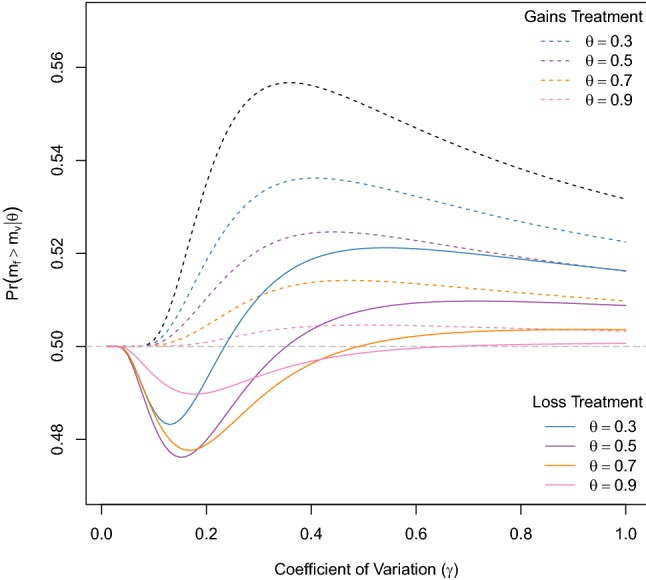
Predictions of the memory model (Eq. 4). Parameters are chosen as in Marsh and Kacelnik (2002), i.e. $a = 2, \,\,b = 6,\,\, f = 4,\,\, p_a = 0.5, \,\,{{s}} = 1$ (gains) and ${{s}} = 7$ (losses), for various values of $\theta$. The dashed black line is the SUT prediction, i.e. $\theta =0$ (colour online)

Figure 1 shows $\Pr (m_f > m_{v} | \theta )$ (Eq. 4) for a range of $\gamma$ values and a set of values for the memory parameter $\theta$. Values for means and probabilities are as in Marsh and Kacelnik (2002) , i.e. $a = 2, b = 6, f = 4$, ${{s}} = 1, 7$, respectively, and $p_a = 0.5 = p_b$.

For $\theta = 0$, we recover the SUT prediction (uppermost curve in Fig. 1) of risk-averse behaviour (preference for the fixed option) for all values of $\gamma$. This prediction does not change with a non-zero memory parameter ($\theta > 0$) in the gains treatment (${{s}} = 1$): our model predicts risk-averse behaviour for all values of $\gamma$. This is qualitatively similar to the SUT prediction, albeit with risk averseness getting weaker with increasing values of $\theta$, tending towards indifference for $\theta = 1$ when only the standard option is compared to itself. In the loss treatment with non-zero $\theta$, on the other hand, we observe both risk-prone and risk-averse behaviour depending on the range of $\gamma$ values. The values of $\gamma$ yielding risk-prone behaviour lie between 0.1 and 0.6, with the upper limit on $\gamma$ increasing from 0.25 for small $\theta$ (0.3) to 0.6 for large $\theta$ (0.9). This prediction deviates from the SUT prediction but reproduces qualitatively the experimental findings. For comparison, the experimentally observed mean values for the probability of choosing the fixed option is $\approx 0.51$ in the gains treatment vs. $\approx 0.40$ in the loss treatment (estimated from Fig.1 in Marsh and Kacelnik (2002)). Note that the observed magnitude of risk-prone behaviour in the loss treatment is greater than that produced by our model.

**Fig. 2:**
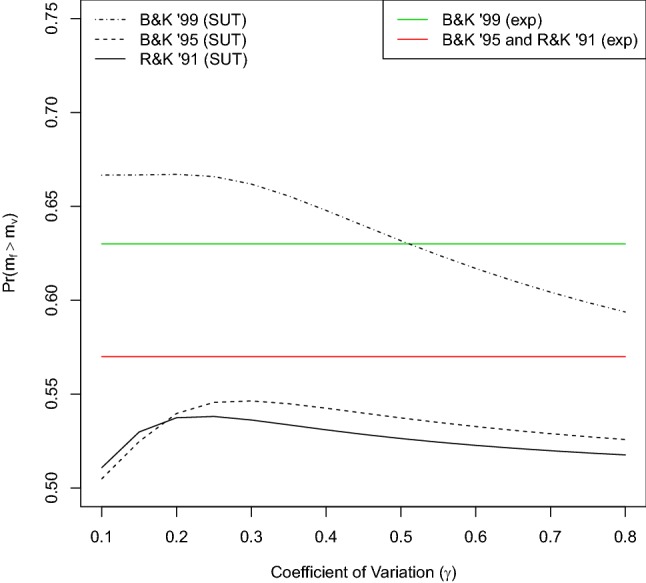
Predictions of SUT (Eq. 2) for the parameter values used in three choice experiments with starlings (Reboreda and Kacelnik 1991; Bateson and Kacelnik 1995; Brito e Abreu and Kacelnik 1999) (see text). The values of the experimental observations are shown as straight lines (colour online)

As can be seen from Fig. 1, we are unable to replicate the observed results unless $\gamma$ is about 0.2. This value is outside the range of 0.3–0.8 reported by Kacelnik and Brito e Abreu Kacelnik and Brito e Abreu (1998). It is, however, compatible with data on starlings. To see this, in Fig. 2 we show predictions of SUT for the probability of choosing the fixed option. We set parameter values of means and probabilities to those used in three different experiments [values given in Reboreda and Kacelnik (1991); Bateson and Kacelnik (1995) and estimated from Fig. 3 in Brito e Abreu and Kacelnik (1999)]. The figure includes the data of the experimental results. For $\gamma = 0.2$, SUT gives a reasonable agreement with the data and for the two lower curves in Fig. 2 (Reboreda and Kacelnik 1991, Bateson and Kacelnik 1995); increasing $\gamma$ to 0.3 or higher brings no improvement. In these cases, the SUT prediction for risk averseness is not particularly sensitive to $\gamma$ between 0.2 and 0.8.

## Discussion

Although it is difficult to give a rigorous definition of framing, the central idea is that it involves presenting essentially the same problem in different ways. This is relatively easy to do in experiments on humans; language allows different descriptions of the same problem. Work on non-humans has to find other ways of framing, e.g. Bhatti et al. (2014); Krupenye et al. (2015); Lakshminarayanan et al. (2011). The study by Marsh and Kacelnik is viewed as a clear example of an experiment on non-human subjects in which the same options are presented as either gains or losses (Krupenye et al. (2015), Kanngiesser and Woike (2016), Krupenye et al. (2016). Although Marsh and Kacelnik establish framing in the general sense that behaviour changes despite the fact that the choice options remain the same, our suggestion is that the value of standard trials changes the context of choice because the decision maker cannot keep the standard trials and the choice trials distinct. Thus, we see the results as illustrating context dependence rather than the more stringent condition of framing in terms of gains and losses, and note that the experimental procedure has similarities to that of Ludvig et al. (2014). Furthermore, our interpretation makes the procedure analogous to experiments which investigate how the relative preference between two options depends on the presence of a third option, e.g. Bateson and Kacelnik (1995), Royle et al. (2008), Monteiro et al. (2013), Lea and Ryan (2015). The fundamental difference is that in these experiments the added option is present in the world and can be chosen, whereas in our interpretation the added option is present only in the animal’s memory. These two cases correspond to what Louie et al. (2015) call spatial context and temporal context, respectively.

## Conclusion

We conclude that the experimental findings on starlings by Marsh and Kacelnik can be explained with a version of SUT which is extended to include the possibility of memory errors. To our mind, this is a more consistent explanation than the concept of framing in terms of gains and losses offers. Our approach makes quantitative predictions that can be checked by estimating $\gamma$. If we assume that decreasing the number of standard trials or making the symbols on the choice keys less similar decreases $\theta$, then we can also make qualitative predictions. Regardless of the quantitative predictions of our model, it is noteworthy that we are able to produce qualitative trends that resemble framing in terms of gains and losses.
